# Human CD133^+^ Renal Progenitor Cells Induce Erythropoietin Production and Limit Fibrosis After Acute Tubular Injury

**DOI:** 10.1038/srep37270

**Published:** 2016-11-17

**Authors:** Shikhar Aggarwal, Cristina Grange, Corinne Iampietro, Giovanni Camussi, Benedetta Bussolati

**Affiliations:** 1 Department of Biotechnology and Health Sciences, Molecular Biotechnology Center, University of Turin, Italy; 2 Department of Medical Sciences, Molecular Biotechnology Center, University of Turin, Italy

## Abstract

Persistent alterations of the renal tissue due to maladaptive repair characterize the outcome of acute kidney injury (AKI), despite a clinical recovery. Acute damage may also limit the renal production of erythropoietin, with impairment of the hemopoietic response to ischemia and possible lack of its reno-protective action. We aimed to evaluate the effect of a cell therapy using human CD133^+^ renal progenitor cells on maladaptive repair and fibrosis following AKI in a model of glycerol-induced rhabdomyolysis. In parallel, we evaluated the effect of CD133^+^ cells on erythropoietin production. Administration of CD133^+^ cells promoted the restoration of the renal tissue, limiting the presence of markers of injury and pro-inflammatory molecules. In addition, it promoted angiogenesis and protected against fibrosis up to day 60. No effect of dermal fibroblasts was observed. Treatment with CD133^+^ cells, but not with PBS or fibroblasts, limited anemia and increased erythropoietin levels both in renal tissue and in circulation. Finally, CD133^+^ cells contributed to the local production of erythropoietin, as observed by detection of circulating human erythropoietin. CD133^+^ cells appear therefore an effective source for cell repair, able to restore renal functions, including erythropoietin release, and to limit long term maldifferentiation and fibrosis.

Acute kidney injury (AKI), described as lowering of glomerular filtration rate and decrease in urine output, affects approximately 10% of hospitalized patients, and its incidence is gradually increasing^1^,^2^. While AKI has been considered for a long time as a completely reversible syndrome, increasing evidence indicate that, in spite of a clinical recovery, it likely results in persistent tissue alterations^3^. In patients, AKI was identified as an independent risk factor for development of chronic kidney disease and end stage renal disease^4^, being the severity of damage the main predictive factor^3^. The mechanisms underlying these clinical results have been depicted in animal models as a process of maladaptive repair, characterized by progressive interstitial fibrosis and loss of function^5^. Maladaptive repair is directly related to persistence of inflammation, loss of vascular density and hypoxia, as well as to cell cycle arrest and senescence of epithelial tubular cells^6^. Molecular alterations after injury involve modulation of several genes with known inflammatory, remodelling and vasoactive activities^7^.

New experimental strategies to promote a correct *restitutio ad integrum*, i. e. the restoration of the original tissue integrity after AKI are therefore needed, possibly including treatment with therapeutic cell sources or reno-protective bioproducts^8^. The population of CD133^+^ renal progenitor cells (CD133^+^ cells) isolated from human tissue has been previously used as a therapeutic cell source in murine models of AKI^9^,^10^,^11^. When injected *in vivo*, CD133^+^ cells, isolated from different nephron segments, ameliorated renal function^9^,^10^,^11^. Using optical imaging, our group also showed that CD133^+^ cells isolated from the renal medulla localized into the injured renal tissue within a short interval of time after injury^10^. Of interest, CD133^+^ cells isolated from renal medulla were previously shown to possess the ability to release erythropoietin (EPO) under hypoxia through a hypoxia inducible factor-2 dependent activation^12^.

EPO has recently emerged as a cyto-protecting factor in organ damage^13^. The administration of recombinant EPO in experimental models of AKI and renal transplant promoted tubular cell survival and tissue vascularization, resulting in improved renal function^14^,^15^,^16^,^17^,^18^,^19^,^20^,^21^ and long term reduction of fibrosis^22^,^23^. EPO-releasing cells of fibroblastic origin have also been proven to be of protection in models of renal injury^24^,^25^. The role of cell therapy on endogenous EPO production however has not been investigated yet.

The present study was designed to investigate the effect of CD133^+^ cells on the long-term alterations of the renal tissue after acute damage in a glycerol-induced AKI model in SCID mice and in particular on the induction of fibrosis. In addition, we aimed to evaluate the effect of CD133^+^ cells on EPO production of both murine and human origin.

## Results

### CD133^+^ cells improved tissue repair and limited fibrosis in AKI SCID mice

**Figure 1:**
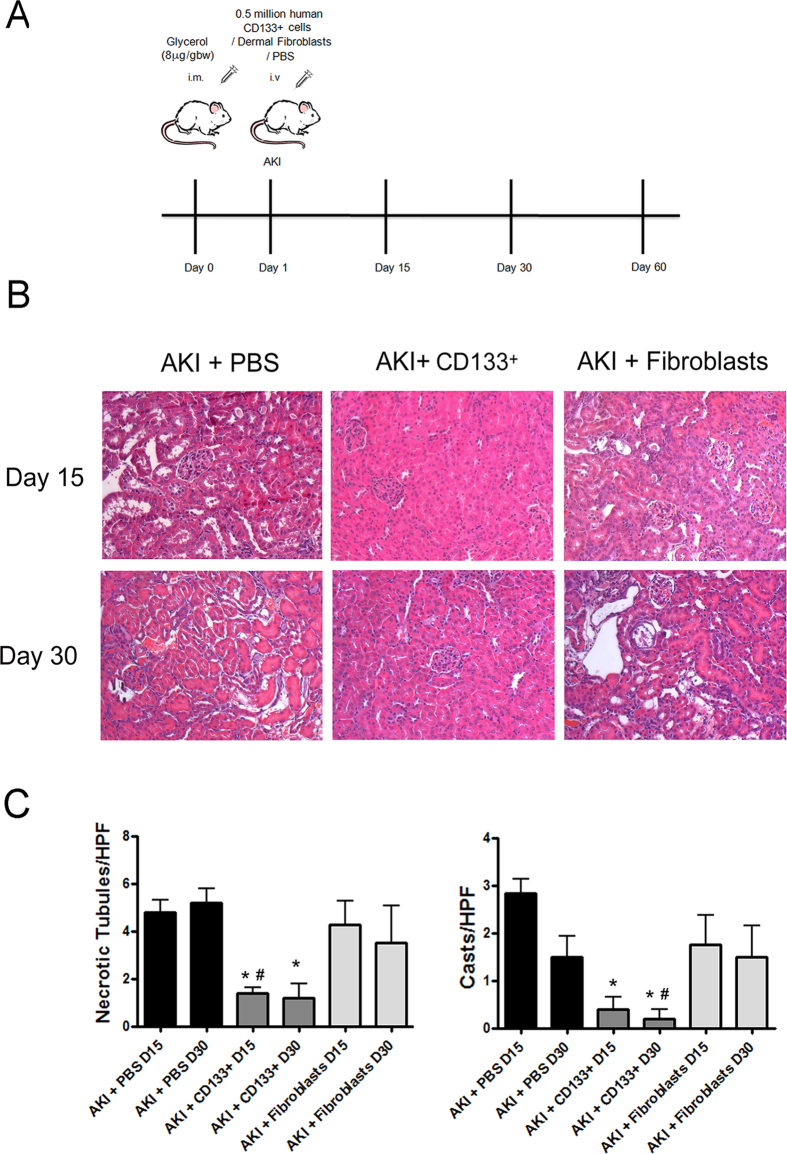
CD133^+^ cells improve renal injury in AKI SCID model. (**A**) Schematic experimental design of the study. (**B**) Representative micrographs of the histological analysis (H&E staining) of renal tissue from AKI mice treated with PBS (vehicle), CD133^+^ cells and dermal fibroblasts at day 15 and 30 after glycerol injection. Original magnification 200×. (**C**) Count of tubular necrosis and tubular hyaline casts at day 15 and 30 after glycerol injection. Data are mean ± SD of the count of 10 fields/section in two sections/mouse. ANOVA with Dunnet’s multicomparison test was performed: *p < 0.01 versus PBS, ^#^p < 0.01 versus fibroblasts.

**Figure 2:**
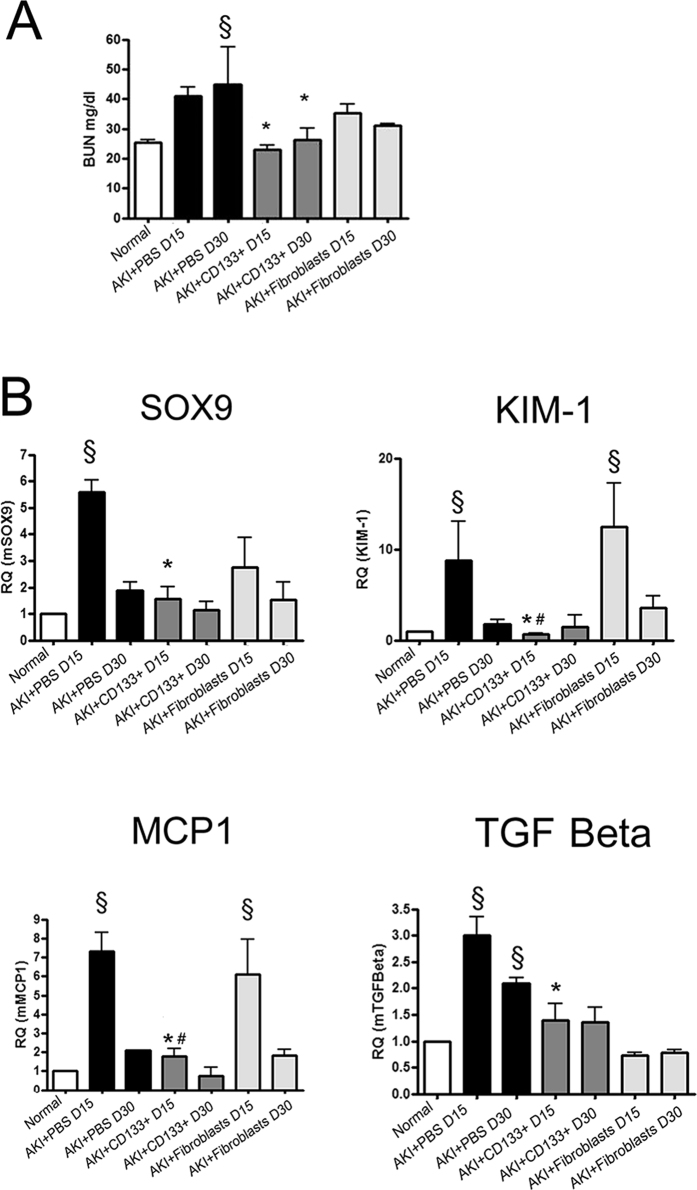
CD133^+^ cells limit injury markers in AKI SCID model. (**A**) Changes in renal function were measured by BUN. Mice treated with CD133^+^ cells showed significantly reduced levels as compared to controls. Data are the mean ± SD of 6 mice/experiment (PBS and CD133^+^ cells) or 3 mice/experiment (Normal or Fibroblasts). (**B**) qRT-PCR analysis of whole mice kidney lysates of normal and AKI mice showing decrease of injury, inflammatory and pro-fibrotic markers in mice that received CD133^+^ cells as compared to PBS or fibroblasts. Data are mean ± SD of three independent experiments and are normalized to GAPDH mRNA and to 1 for normal and expressed as relative quantification (RQ). ANOVA with Newman Keuls’ multicomparison test was performed: ^§^p < 0.01 versus Normal; *p < 0.01 versus PBS, ^#^p < 0.01 versus fibroblasts.

A model of glycerol-induced rhabdomyolysis was used to evaluate the effects of CD133^+^ cell administration on persistence of molecular alterations and on fibrosis development after AKI (Fig. 1A). This model is characterized by a severe kidney injury and rapid loss of renal function (24 hour BUN: 120±14 mg/dl, n = 6) and subsequent progressive repair^10^. After 15 and 30 days, AKI mice did not completely recover renal damage, as evaluated by persistence of hyaline casts and necrotic/dilated tubules (Fig. 1B,C) and of BUN at levels higher than basal (Fig. 2A). Moreover, in renal tissue of AKI mice, expression of markers of tubular cell injury and stress, inflammation and fibrosis were observed (Fig. 2). In particular, mice showed increased expression of SOX9, kidney injury marker KIM-1, and of the pro-inflammatory marker MCP-1 (Fig. 2B) at day 15. These markers returned at basal level at day 30 (Fig. 2) and 60 (not shown). As detailed in the experimental design (Fig. 1A), 500,000 CD133^+^ cells isolated from human renal medulla and characterized as described^26^ were injected i.v. in SCID mice one day after glycerol-induced injury. Human dermal fibroblasts (500,000 cells) were used as control. Mice injected with CD133^+^ cells exhibited improvement in the renal tissue, with absence of casts, necrosis of tubular epithelial cells and tubule dilation and recovery of tissue architecture (Fig. 1B,C). In parallel, renal function at day 15 and 30 was normal (Fig. 2A). At variance, injection of fibroblasts did not ameliorate the renal structural modification and renal function in AKI mice (Figs 1 and 2). In addition, SOX-9, KIM-1 and MCP-1 were significantly reduced in kidneys of mice treated with CD133^+^ cells as compared to controls at day 15. No effect of fibroblast administration was observed, in terms of renal function, histological alterations and injury marker expression (Figs 1 and 2). In addition, the pro-fibrotic marker TGF-β which was elevated up to day 30 in control mice, showed reduced levels in CD133^+^ cell treated mice, as well as in fibroblast treated mice (Fig. 2B).

**Figure 3:**
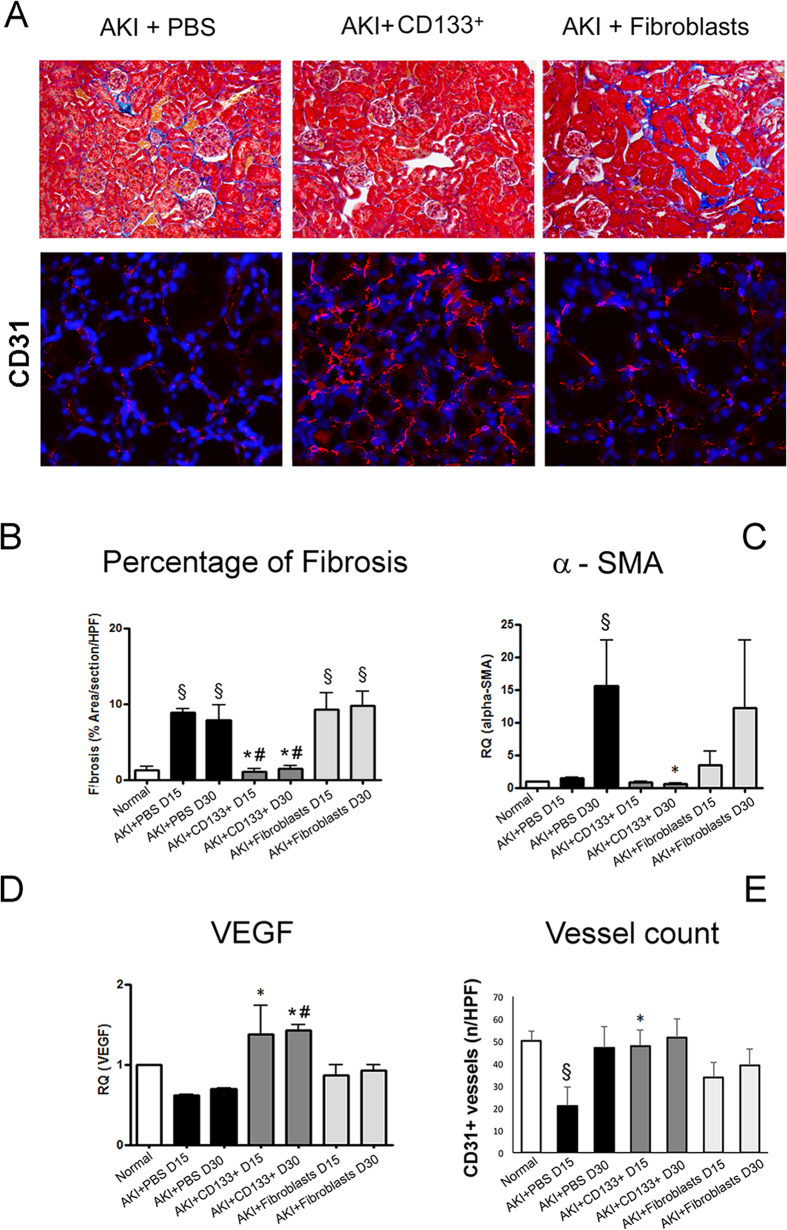
CD133^+^ cells protect against fibrosis and promote vascularization in AKI SCID model. (**A**) Representative micrographs showing renal tissues of mice treated with PBS, CD133^+^ and dermal fibroblasts at day 15 after glycerol injection. Upper panels: Masson’s trichrome staining showing collagen in blue and erythrocytes in yellow. Lower panels: Immunofluorescence staining of CD31 positive vessels, shown in red. Nuclei were counterstained with DAPI (blue). Original magnification: upper panels: 200×; lower panel: 400×. (**B**) Quantification of fibrosis in normal and AKI mice performed by count of collagen area (blue colour) in the interstitium by Image J software on 10 fields/section in two sections/mouse. (**C,D**) qPCR analysis of whole kidney lysates of normal and AKI mice for α-SMA and VEGF. Data are mean ± SD of three independent experiments and are normalized to GAPDH mRNA and to 1 for normal and expressed as relative quantification (RQ). (**E**) Count of CD31^+^ structures of normal and AKI mice kidney tissue performed on 10 fields/section in two sections/mouse. ANOVA with Newman Keuls’ multicomparison test was performed: ^§^p < 0.01 versus Normal; *p < 0.01 versus PBS, ^#^p < 0.01 versus fibroblasts.

**Figure 4:**
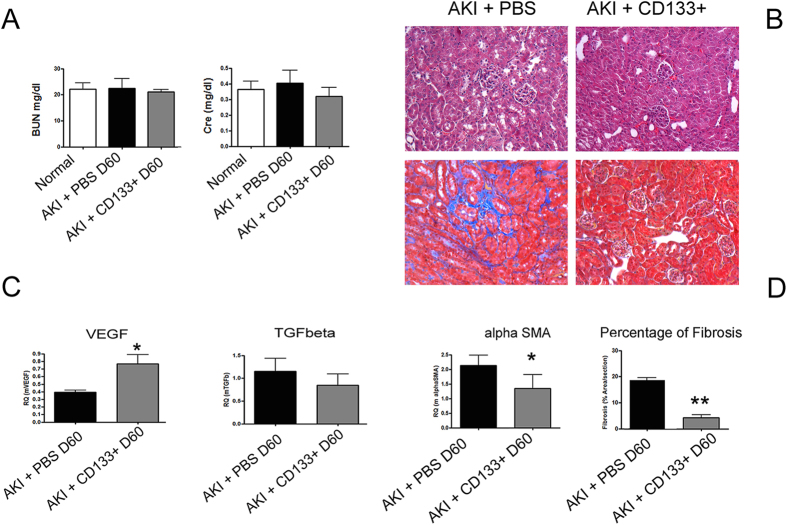
Effect of CD133^+^ cells on fibrosis and vascularization at day 60. (**A**) Plasma analysis for BUN and creatinine in AKI mice showing normalized renal function at day 60 after injury. (**B**) Representative micrographs of H&E and Masson’s trichromic staining of renal tissue in mice treated with PBS or CD133^+^ cells at day 60 after glycerol injection. Original magnification 200×. (**C**) qPCR analysis of whole kidney lysates for the pro-angiogenic factor VEGF and for the fibrotic factors TGF-β and α-SMA. Data are mean ± SD of three independent experiments and are normalized to GAPDH mRNA and to 1 for normal and expressed as relative quantification (RQ). (**D**) Quantification of fibrosis in the interstitium by Image J software was performed on 10 fields/section in two sections/mouse. T-test: **p < 0.01; * < 0.05.

We next assessed the effect of CD133^+^ cells on development of fibrosis, as evaluated by Massons Trichrome staining and α-SMA detection in renal tissue. In AKI control mice, a moderate degree of fibrosis was observed at day 15 and 30 after damage (Fig. 3A). Mice group receiving CD133^+^ cells showed significantly reduced fibrosis both at day 15 and day 30 as compared to control mice (Fig. 3A). In addition, qRT-PCT analysis of whole kidney lysate also showed reduced levels of α-SMA expression in mice that received CD133^+^ cells as compared to controls (Fig. 3B). No limiting effect on fibrosis was observed in mice receiving fibroblasts (Fig. 3A,B). As development of fibrosis is associated with vessel rarefaction^27^,^28^, we next evaluated tissue vascularization. We found improved vascularization in terms of increased expression of the angiogenesis factor VEGF as compared to mice treated with PBS only or fibroblasts (Fig. 3D). In parallel, the number of CD31^+^ peritubular capillaries was restored in mice receiving CD133^+^ cells at day 15 (Fig. 3A,E). We further analyzed the effect of the CD133^+^ cells up to day 60 on the progression of AKI. At day 60, AKI mice showed complete recovery of renal function, as mice that received CD133^+^ cells did (Fig. 4A). However, control mice progressed towards fibrosis, whereas mice injected with CD133^+^ cells showed significant lower presence of fibrosis, evaluated by histological analysis and α-SMA levels (Fig. 4B,C). Interestingly, mice that received CD133^+^ cells maintained significantly higher renal levels of VEGF and lower levels of TGF-β (Fig. 4C).

### CD133^+^ cells localized into interstitium and tubules of injured kidneys

**Figure 5:**
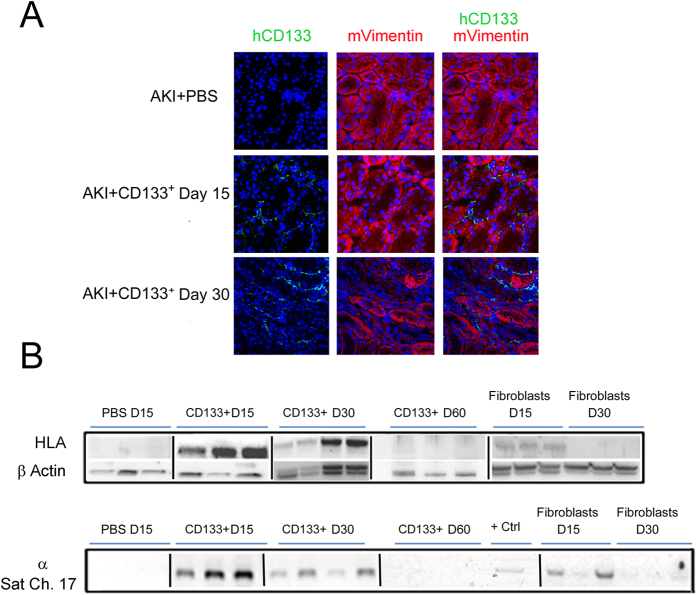
Intravenously injected CD133^+^ cells localization in kidneys of AKI SCID mice. (**A**) Representative confocal micrographs showing the presence of CD133^+^ cells localized in the interstitium and tubules within the kidney of AKI mice at day 15 and day 30 after damage as evaluated by human CD133 (Green) and mouse Vimentin (red). Nuclei were counter-stained with DAPI (blue). Original magnification 400×. (**B**) HLA protein and whole genome DNA analysis (α-Sat ch17) of mice kidneys at day 15, day 30 and day 60 after glycerol injection. CD133^+^ cells were present up to day 30 as shown by immunofluorescence or protein/DNA analysis, whereas dermal fibroblasts were only detected at day 15. + Ctrl: positive control using human CD133^+^ cells. Lanes run on different gels are separated by a dark line.

We previously reported the bio-distribution of injected CD133^+^ cells after 3 and 5 days in AKI mice. Fluorescence analysis of dissected organs showed comparable cell accumulation in lungs, liver and kidneys^10^. Here, we evaluated the localization and persistence of CD133^+^ cells after 15 and 30 days. Immunofluorescence staining using human CD133 showed the engraftment of CD133^+^ cells within the renal tissue (Fig. 5A). Mouse Vimentin was used to stain filament structures in recovering tubules. Cells were both present in renal interstitial tissue and integrated within tubular structures, within renal cortex. HLA protein detection and DNA analysis for human alpha-satellite Cr-17 confirmed persistence of CD133^+^ cells into renal tissue upto day 30 (Fig. 5B). In addition, protein and DNA analysis also shown presence of CD133^+^ cells in lungs and liver at day 15 (Supplemental Figure 1). In contrast, at day 30, dermal fibroblasts could not be detected within the renal tissue (Fig. 5B) nor in lungs or liver (Supplemental Figure 1). At 60 days after injection however, human cells were not detectable in murine tissues by DNA analysis and at very low level by Western Blot for HLA (Fig. 5B).

### CD133^+^ cells stimulate EPO production (of both human and mice origin) *in vivo*

**Figure 6:**
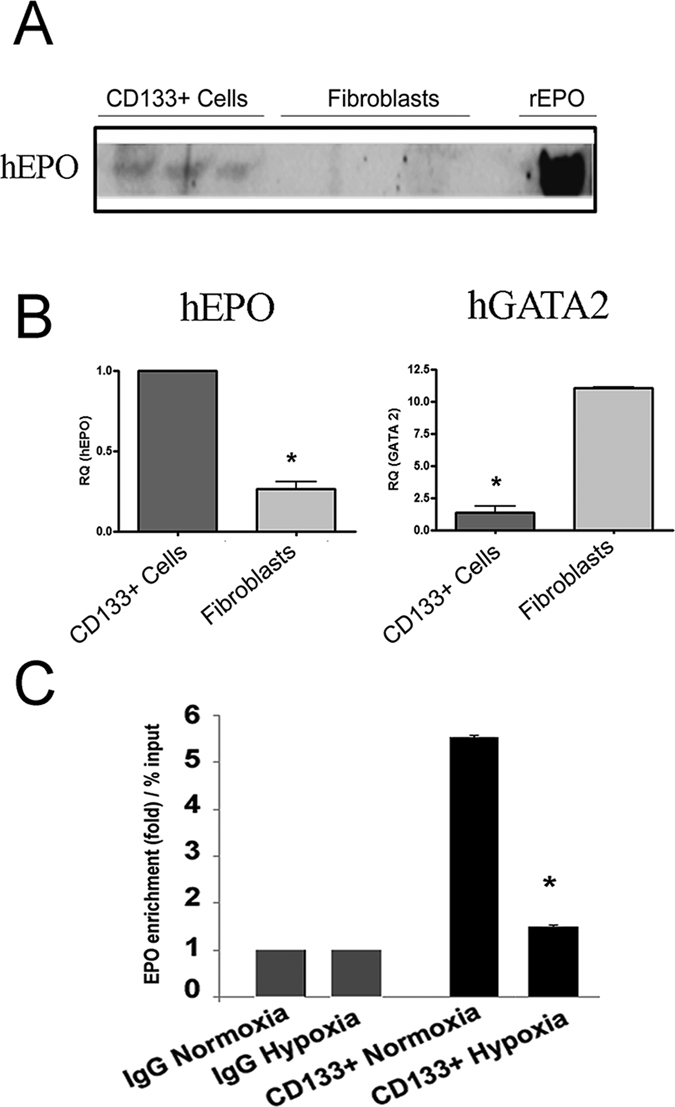
EPO analysis in CD133^+^ cells and dermal fibroblasts. (**A**) Micrographs showing Western Blot analysis of hEPO in the supernatant cultures of CD133^+^ cells as compared to fibroblasts (3 representative cell lines). Recombinant EPO (rEPO) was loaded as control. (**B**) Quantitative RT-PCR analysis showing increased mRNA levels of human EPO and decreased GATA2 expression in CD133^+^ cells as compared to fibroblasts. Data are the mean ± SD of three experiments. Data are normalized to GAPDH mRNA and to 1 for CD133^+^ cells and expressed as relative quantification (RQ). (**C**) ChIP analysis of binding of GATA2 at EPO transcriptional motif in CD133^+^ cells in normoxia or hypoxia. Hypoxia reduced the binding of GATA2 by five folds in CD133^+^ cells. All data are means ± SD of 3 different experiments. T-test: *p < 0.01.

We have previously reported EPO production by CD133^+^ cells from the medulla under hypoxia in a HIF-2 dependent manner^12^. In culture, CD133^+^ cells but not fibroblasts secreted EPO (Fig. 6A). This was confirmed by higher expression of EPO mRNA and lower expression of the EPO transcriptional repressor GATA2 in CD133^+^ cells as compared to fibroblasts, as analysed by qRT-PCR (Fig. 6B). In addition, GATA2 binding to the EPO transcriptional motif was significantly downregulated by hypoxia as evaluated by ChIP analysis (Fig. 6C).

**Figure 7:**
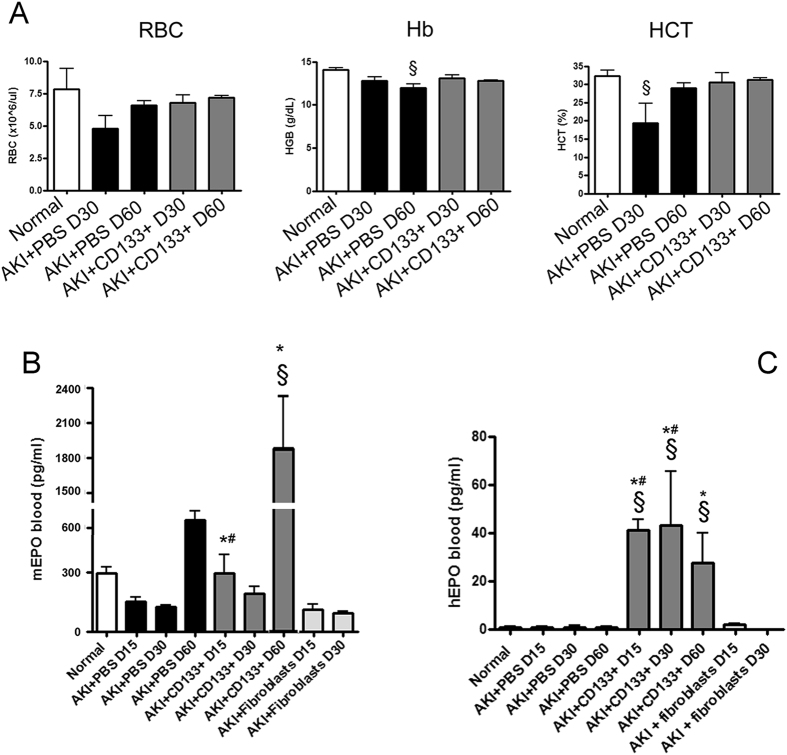
Analysis of AKI mice blood. (**A**) Whole blood analysis of mice (normal and AKI mice) at day 30 and day 60, showing levels of red blood cells (RBC), haemoglobin (Hb) and haematocrit (HCT). Data are mean ± SD of three different mice per group. Newman Keuls’ multicomparison test was performed: ^§^p < 0.01 versus Normal. (**B,C**) Detection of murine and human EPO in plasma of normal and AKI mice treated with PBS, CD133^+^ cells or dermal fibroblasts at day 15, 30 and 60 after injury. Data are mean ± SD of three experiments analysing at least three different mice per group. Newman Keuls’ multicomparison test was performed: ^§^p < 0.01 versus Normal; *p < 0.01 versus PBS, ^#^p < 0.01 versus fibroblasts.

Therefore, we investigated the effect of CD133^+^ cells on EPO *in vivo* in AKI mice. Previous studies showed that haemoglobin levels are reduced in animals with glycerol-induced AKI in respect to control^14^. We also found that AKI mice had a mild decrease in the haematocrit level, haemoglobin and erythrocyte count at day 30 that was absent in CD133^+^ cell-treated mice (Fig. 7A). In parallel, we observed that AKI mice showed a significant decrease of circulating EPO at day 15 and 30 to increase at day 60 (Fig. 7B), as evaluated by ELISA. Similar lower levels of circulating EPO were observed in fibroblast-treated animals (Fig. 7B). In CD133^+^ cell-treated mice, circulating levels of murine EPO were comparable to control (Fig. 7B). Interestingly, at day 60, the level of mouse EPO increased in CD133^+^ cell injected mice as compared to controls, suggesting that CD133^+^ cells stimulated local EPO production (Fig. 7B). In addition, circulating amounts of human EPO, although at low levels (around 100 fold lower) as compared to murine EPO, were detected at day 15, 30 and 60 (Fig. 7B), as assessed by a human EPO specific ELISA.

**Figure 8:**
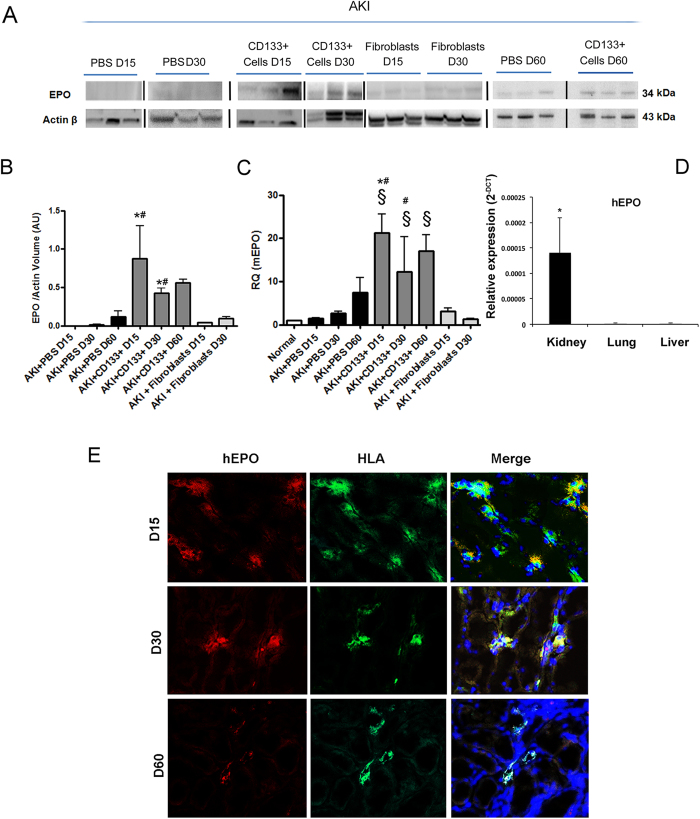
Effect of CD133^+^ cells on human and murine EPO. (**A,B**) Western blot analysis and quantification of EPO protein in whole kidney lysate of mice treated with PBS, CD133^+^ cells or dermal fibroblasts at day 15, 30 and 60 after injury. Data are mean ± SD of three experiments analysing at least three different mice per group. Lanes run on different gels are separated by a dark line. (**C**) Quantitative RT-PCR analysis of mice EPO in whole kidney lysate of normal and AKI mice. Increased EPO levels are observed in CD133^+^ cell treated mice as compared to controls or fibroblasts. Data are normalized to GAPDH mRNA and to control and are expressed as mean ± SD of three to six different mice per group. Newman Keuls’ multicomparison test was performed: ^§^p < 0.01 versus Normal; *p < 0.01 versus PBS, ^#^p < 0.01 versus fibroblasts. (**D**) Quantitative RT-PCR analysis of human EPO in whole lysate of kidneys, lungs and livers of CD133-treated AKI mice at day 15. EPO levels were undetectable in lungs and liver. Data are reported as the relative expression (2^−ΔCT^). GAPDH was used as housekeeping gene. N = three mice. (**E**) Representative confocal micrographs showing the presence of human EPO (red) and HLA positive cells (green) within the kidney of AKI mice treated with CD133^+^ at day 15, 30 and day 60 after damage. Nuclei were counter-stained with DAPI (blue). Original magnification 400×.

The effect on EPO synthesis was further confirmed by the presence of higher levels of EPO protein (of mouse and human origin) in the whole kidney lysate of mice that received CD133^+^ cells as compared to control (Fig. 8A and B). Murine EPO was significantly increased in kidneys of cell-treated mice (Fig. 8C). Human EPO mRNA was only detectable, using human specific primers, within the renal tissue and not in liver or lungs (Fig. 8D). Immunofluorescence analysis on renal tissue identified that HLA^+^ cells present within tubular interstitium also expressed the human EPO protein (Fig. 8E). These data altogether indicate a prominent effect of CD133^+^ cells on regulating EPO levels in the kidney after AKI.

## Discussion

In our study, we evaluated the functional role and fate of human adult CD133^+^ renal progenitor cells derived from medulla region of kidney in renal repair using an established animal model of glycerol-induced AKI in SCID mice followed up to day 60. Our data showed that adult human CD133^+^ cells favoured the restoration of the renal tissue, limiting the presence of pro-inflammatory and pro-fibrotic molecules, promoting angiogenesis and protecting against fibrosis in AKI mice, as compared to control group with PBS or fibroblasts cells. In addition, CD133^+^ cells promoted the endogenous production of EPO, limiting its transient reduction and mice anaemia after AKI, and contributed to the release of human EPO in the mouse circulation.

A number of studies showed a protective effect of adult human renal cells in tissue protection and functional repair in AKI, including CD133 expressing cells^8^. An effect on the reduction of early tissue fibrosis (up to day 14) was also shown using CD133^+^ embryonic renal cells^29^. In addition, adult mature renal cells and EPO-releasing fibroblasts have been proven to ameliorate renal function in models of chronic renal damage^24^,^25^. In the present study, we investigated the effect of CD133^+^ cells during resolution of AKI, focusing not only on markers of renal injury, but also on molecular alterations involved in the persistence of damage as well as on maladaptive repair, leading to fibrosis and possibly to chronic injury. Here we found an increase of all the markers of stress and injury, inflammatory and pro-fibrotic environment. SOX9 and KIM-1 upregulation following AKI were shown to represent not only markers of injury, but also of unresolved repair^30^. Interestingly, CD133 injection was shown to limit all the observed alterations occurring in the renal tissue, together with acceleration of recovery. A mild effect on renal function was surprisingly also induced by fibroblast administration. However, fibroblasts did not protect against maladaptive repair and fibrosis.

The mechanism involved in the repair after human renal cell administration can be ascribed to integration into the renal tubules and in parallel to a paracrine mechanism based on reno-protective molecules^8^. We here observed a persistence of CD133^+^ cells up to 30 days within the tissue that may possibly underline a transient integration within renal structures as well as a paracrine mechanism. In addition the release of pro-active renal protective factors from distal sites may also be involved, as cell also localized in organs such as lungs and liver.

Among the possible paracrine effects of CD133^+^ cells, we focused our attention on EPO levels for two main reasons. First, decreased EPO levels were reported after AKI in experimental animals and in patients^31^,^32^,^33^,^34^ indicating a defect in the response of EPO-releasing cells to acute toxic/ischemic damage^35^. Accordingly, we found that AKI mice showed a transient reduction of EPO levels and a light anaemia. This can possibly be ascribed to increased levels of local pro-fibrotic and pro-inflammatory mediators, as proinflammatory cytokines occurring in AKI may activate the EPO transcriptional repressors GATA2 and NF-kB^36^,^37^. In addition, pro-fibrotic mechanisms may also promote epigenetic alterations (like hypermethylation) in the EPO promoter, inducing EPO repression and leading in the same time to maldifferentiation of EPO producing cells into myofibroblasts^38^,^39^, as recently described in chronic kidney disease^39^. In addition, CD133^+^ cells may directly favour survival of EPO-releasing nephron cells, reported to be damaged by toxic mediators^40^.

Secondly, EPO is gaining increased attention in AKI due to its cyto-protective effects proven in a number of animal models^14^,^15^,^16^,^17^,^18^,^19^, being administration to patients still under investigation in clinical trials^13^,^41^. In the renal tissue, EPO may induce protective intracellular pathways, such as JAK2, PI3K/AKT and eNOS^42^, reduce expression of proinflammatory cytokines and promote angiogenesis^43^.

We here found that CD133^+^ cells promoted the synthesis of murine EPO within the kidney and its release within the circulation. The mechanism involved in direct EPO stimulation is unclear, and could be possibly associated with the reduction of tissue damage. However, a direct and specific effect of progenitor cell derived factors could be also postulated. Indeed, a similar effect on local EPO synthesis within the neural tissue has been recently reported after mesenchymal stem cell administration in a model of acute brain damage^44^.

EPO induction could be beneficial for its hemopoietic effect, as shown by lack of haematocrit drop. Beside that, increased EPO levels within the kidney may exert a cytoprotective effect. In agreement, previous data showed a beneficial effect of mesenchymal stem cells genetically modified to release EPO or treated with recombinant EPO in kidney injury models^24^,^25^,^45^.

In addition, we have also found that injected CD133^+^ cells transiently released EPO *in vivo*, as shown by detection of circulating human EPO in the plasma of AKI mice. CD133^+^ cells were previously shown to release EPO under hypoxia^12^, and we have also here confirmed their capacity with respect to human fibroblasts, that were used as controls in our study. The role of CD133^+^ cells in physiological EPO synthesis within the nephron is unclear, as EPO production has been reported to occur in the peritubular interstitial fibroblasts^46^,^47^,^48^. However, recent data from the literature showed that whereas murine peritubular fibroblasts are mainly responsible for EPO production after hypoxic stimulation, cells within the nephron provide basal EPO release under normal homeostasis^49^. EPO production by renal cells is limited by transcriptional EPO repressor GATA2, known to be acquired by differentiated tubular cells^37^. However, as CD133^+^ scattered cells within the nephron are characterized by a poorly differentiated phenotype^9^,^50^, it is conceivable that they could release EPO also *in vivo*. Accordingly, we found that CD133^+^ cells presented low levels of GATA2 in respect to fibroblast, and GATA2 binding to the EPO gene was further downregulated by hypoxia.

In summary, in our model, CD133^+^ cells showed the ability to promote the release of the hemopoietic and renoprotective hormone EPO *in vivo* and abrogated EPO reduction occurring after AKI by stimulating the release of the EPO of both mice and human origin. Taking all these data together, CD133^+^ cells appear as a promising source for cell therapy to promote the acceleration of renal recovery and limit unresolved/maladaptive repair.

## Methods

### Cells

CD133^+^ cells were isolated from human adult renal medulla as described earlier^27^. Methodology was carried out in accordance with the guidelines approved by the local Ethical Review Board of “Azienda Ospedaliera Città della salute e della Scienza” and all patients signed the informed consent. Briefly, renal medulla tissues were obtained from surgically removed kidneys from the patients. Tissue samples were cut into small pieces and digested in 0.1% Collagenase type I (Sigma-Aldrich) for 35 min at 37 °C. Afterwards, fragmented collagenased tissue was passed through a graded series of meshes to obtain a cell suspension. Cells were re-suspended in expansion medium (endothelial basal medium (EBM) plus supplement kit; Cambrex BioScience) without serum addition at a density of 1.0 × 10^4^ viable cells per cm^2^ and cultured. Cells were characterized on the basis of the expression of a panel of renal and mesenchymal markers, and co-expressed CD133 and CD24, previously to specifically stain renal stem cell^51^ (Supplementary Table 1 and Supplementary Figure 2). Human dermal skin fibroblasts (Lonza) were used as control cell line and were maintained in culture in FBM media with supplements (Cambrex BioScience) upto passage 4.

### Animals and experimental set up

Immunodeficient SCID mice were maintained as per the institute ethical guidelines for the use and care of laboratory animals. SCID mice were kept under well-controlled conditions of temperature (22±2 °C), humidity (55±5%) and a 12 h/12 h light-dark cycle with access to food and water ad libitum. The protocol was approved by the Bioethical Committee of the University of Torino (no. 7/2014/B2) and by the Italian Ministry of Health (n. 274/2015PR), and conducted in accordance with the National Institutes of Health Guide for the Care and Use of Laboratory Animals. AKI was created in male SCID mice (7–8 weeks old) by intramuscular injection of a half dose of 8 mg/kg of 50% solution of hypertonic glycerol (Sigma) in water in each leg of the mice in anaesthetic condition. Five hundred thousand cells/150 μl injection volume or the same volume of PBS (n = 10 mice/group for PBS and CD133^+^ cells; 3 mice/group for fibroblast treatment) were injected per mice intravenously into the tail vein one day after injury and mice were monitored for health and sickness upto day 60. After sacrifice, organs (kidneys, lungs and livers) were recovered for analysis. In particular, one and half of mouse kidneys were meshed with syringe head over 40 μm filters in PBS, washed and tissue lysates were obtained. Cell homogenates were then immediately processed for different analysis of protein, DNA or RNA analysis. Other half of the kidney was used for paraffin embedded sections or cryo-sections.

### Morphological Studies

For renal histology, 5 μm thick paraffin kidney sections were routinely stained with hematoxylin and eosin (Merck) or Masson’s Trichrome blue (Dako) as per manufacture’s protocol. Luminal hyaline casts and cell lose (denudation of tubular basement membrane) were assessed in non-overlapping fields (up to 10 for each section) using a 20× objective (high power field, HPF). Number of casts and tubular profiles showing necrosis was recorded in a single blind fashion as previously reported^10^. Tissue fibrosis was measured in kidney sections using Masson’s Trichrome blue. Presence of blue colour represented deposition of collagen fibres between tubules and quantified by Image J software.

### Immunofluorescence analysis

Confocal microscopy analysis was performed on frozen sections for the detection of human CD133^+^ cells. Sections were labelled with the following primary antibodies: anti-CD133 mAb (1:10) (clone 293C3, Miltenyi), anti-human EPO mAb (1:200) (TermoFisher), anti-vimentin goat Ab (1:200) (Sigma) and anti-HLA rabbit Ab (1:100) (Santa Cruz Biotechnology). Rabbit anti-mouse FITC or chicken anti-goat PE or anti-rabbit PE Abs (1:500) (Molecular Probes) was used as secondary antibodies. Hoechst 33258 dye (Sigma) was added for nuclear staining.

Vessels were counted in the kidney sections stained with CD31 Ab (1:100, Santa Cruz). CD31^+^ structures were counted in non-overlapping fields (up to 10 for each section) for at least 3 mice per group using a 40× objective (HPF) in a single blind manner. Confocal microscopy analysis was performed using a Zeiss LSM 5 Pascal Model Confocal Microscope (Carl Zeiss International).

### RNA analysis

Total RNA was isolated from different cell preparations using the TRIzol® Reagent RNA Isolation protocol (Ambion, life technologies). Briefly, cells were lysed directly in a culture dish by adding 1 mL TRIzol® per 10 cm^2^ of culture dish surface area. To allow phase separation, 0.2 ml of chloroform per 1 ml of TRIZOL Reagent was added to lysates; lysates were then subjected to vortex and centrifugation at 12000 rpm at 4 °C for 15 minutes. The upper aqueous phase containing RNA was transferred in fresh tubes and isopropanol (0,5 ml of isopropanol per 1 mL TRIZOL Reagent used in the initial homogenization) was added to allow RNA precipitation. Samples were then incubated at RT for 10 minutes and subjected to centrifugation at 12000 rpm for 10 minutes. After supernatant removal, RNA pellets were washed with 70% ethanol, left to air dry for 10–15 minutes and resuspended in 20–50 μl of RNase-free water. RNA was then quantified spectrophotometrically (Nanodrop ND-1000, Wilmington DE). For gene expression analysis, quantitative real-time PCR (qRT-PCR) was performed. Briefly, first-strand cDNA was produced from 200 ng of total RNA using the High Capacity cDNA Reverse Transcription Kit (Applied Biosystems). qRT-PCR experiments were performed in 20 μl reaction mixtures containing 10 ng of cDNA template, the sequence-specific oligonucleotide primers (MWG-Biotech AG) and the Power SYBR® Green PCR Master Mix (Applied Biosystems). β-actin or GAPDH mRNA were used to normalize RNA inputs. Fold change expression with respect to control was calculated for all samples.

### Sequence-specific oligonucleotide primers

Human β-actin: forward, 5′-TGA AGA TCA AGA TCA TTG CTC CTC-3′ and reverse, 5′-CAC ATC TGC TGG AAG GTG GAC-3′; Human EPO 5′-GGAAAGTGTCAGCAGTGATTGTTC-3′, 5′-AGCCCAGAAGGAAGCCAT CT-3′; human GATA2 forward 5′-ACTGACGGAGAGCATGAAGAT-3′ and reverse 5′ CCGGCACATAGGAGGGGTA-3′; mSOX9 forward: 5′-AGTACCCGCATCTGCACAAC-3′, reverse: 5′-ACGAAGGGTCTCTTCTCGCT-3′; mKIM-1 forward: 5′-ATGAATCAGATTCAAGTCTTC-3′, reverse: 5′-TCTGGTTTGTGAGTCCATGTG-3′; mTGF-β forward: 5′-GCAACAATTCCTGGCGTTACC-3′, reverse: 5′-CGAAAGCCCTGTATTCCGTCT-3′; mαSMA forward: 5′-CTGACAGAGGCACCACTGAA-3′, reverse: 5′-CATCTCCAGAGTCCAGCACA-3′; mMCP-1 forward: 5′-TGCATCTGCCCTAAGGTCTTC-3′, reverse: 5′-AAGTGCTTGAGGTGGTTGTGG-3′; mVEGF forward: 5′-GAGCAGAAGTCCCATGAAGTGA-3′, reverse 5′-CACAGGACGGCTTGAAGATGT-3′; mEPO forward 5′-ACTCTCCTTGCTACTGATTCCT-3′ and reverse 5′-ATCGTGACATTTTCTGCCTCC-3′; mGAPDH, forward 5′-TGTCAAGCTCATTTCCTGGTATGA-3′, reverse 5′-TCTTACTCCTTGGAGGCCATGT-3′.

### Protein analysis

For protein analysis, cell homogenates obtained from animal-tissue lysates were lysed at 4° C for 30 min in RIPA buffer (20 nM Tris-HCL, 150 nM NaCl, 1% deoxycholate, 0.1% SDS, 1% Triton X-100, pH 7.8) supplemented with protease and phosphatase inhibitors cocktail and PMSF (Sigma). For *in vitro* culture’s supernatant analysis, supernatants were collected from CD133^+^ cell and fibroblasts cell cultures and concentrated using ultracel-10 K centrifugal filters (Millipore). 30 μg of protein, determined by the Bradford’s method, were, then, run on 4–20% Mini-Protean TGX^TM^ Gel (Bio Rad) and blotted onto PVDF membrane filters with iBlot (Life Technologies). The transfer of proteins onto a PVDF membrane was performed in a 7 minutes transfer program of the iBlot™ Dry Blotting System (Life Technology). Rabbit polyclonal primary antibodies against HLA (MHC class 1), EPO and β-actin (all from Santa Cruz Biotechnogy) were used.

### Whole genome DNA analysis

Whole genome DNA is extracted from the frozen cells isolated from animal tissues (kidneys, lungs and livers) using DNA isolation kit (Qiagen), quantified and 200 ng of DNA is PCR amplified for human specific DNA repeat α-Satellite Chromosome 17 (1171 bp fragment) using the following primers: forward 5′-ACACTCTTTTTGCAGGATCTA-3′ and reverse 5′-AGCAATGTGAAACTCTGGGA-3′ as published earlier^52^. The PCR samples were then analysed on Agarose gel electrophoresis and analysed using Gel-Doc system (BioRad). DNA isolated from human CD133^+^ cells were used as positive control and water used as negative control.

### ChIP assay

ChIP assay was performed to analyse the binding of transcription factor GATA2 with EPO gene in human CD133^+^ cells, as previously described with some modifications^53^. Briefly, CD133^+^ cells were cultured in normoxia or hypoxia (1% O_2_) for 48 hours and harvested. Proteins and DNA were cross-linked by incubating 3 × 10^6^ cells in 1% (v/v) formaldehyde for 10 min. Genomic material was then obtained after sonification of cells and 10% of material ‘termed input’ was freezed at −20°C for later use. For immunoprecipitation, GATA2 antibody (Abcam, 5 μg) was used. Rabbit IgG (Abcam, 5 μg) was used as control antibody. The chromatin was immunoprecipitated by incubation with 100 μl of Protein A-Sepharose beads. Immunoprecipitates were washed, DNA was eluted (Qiagen) and the cross-linking was reversed by incubating samples overnight at 65 °C. DNA was purified (QIAprep Spin Miniprep kit, Qiagen) and eluted in 40 μl of elution buffer. For amplification by PCR, one third of input DNA (diluted in water), 1 μl of the “IgG antibody” sample, or 1 μl of immunoprecipitated DNA were mixed with 0.1 μl of primers (forward and reverse each, 10 μM) and 10 μl of PCR buffer (2X) to a total volume of 20 μl. The following primer pair was used: Epo 5′-flanking region: 5′ primer, 5′-ACTCAGCAACCCAGGCATCTCTGA-3′; 3′ primer, 5′-CGACCCCTCACGCACACAGCCTCT-3′; All samples were processed in the same PCR reaction and performed in triplicate.

### Blood analysis for renal function

Blood samples for measurement of serum creatinine and blood urea nitrogen (BUN) were collected at day 15, day 30 and day 60. Serum creatinine was measured using a colorimetric microplate assay based on the Jaffe reaction (Quantichrom Creatinine Assay, BioAssay Systems). BUN levels were measured by commercially available kit as per the manufacturer’s guidelines (Quantichrom Urea Assay, BioAssay Systems).

### Blood analysis for EPO and routine parameters

Human and mouse EPO protein in mice plasma was measured by Platinum ELISA short incubation kit (eBioscience) and Quantikine ELISA (R&D Systems) respectively, according to the manufacturer’s recommendations. Specificity of the human ELISA kit was confirmed by lack of reactivity in plasma of SCID, B6 and FVB mice (not shown). Blood parameters (hematocrit, red blood cell (RBC) counts and haemoglobin) were measured by routine blood analysis as per the instructional manual of BC-5300 VET automatic Hematology Analyzer (MindRay).

### Statistical analysis

Statistical analysis was performed using the Student t test, or ANOVA with Dunnet’s or Newman Keuls’ multi-comparison tests, as appropriate. A p value of < 0.05 was considered significant.

## Additional Information

**How to cite this article**: Aggarwal, S. *et al.* Human CD133^+^ Renal Progenitor Cells Induce Erythropoietin Production and Limit Fibrosis After Acute Tubular Injury *Sci. Rep.* **6**, 37270; doi: 10.1038/srep37270 (2016).

**Publisher’s note:** Springer Nature remains neutral with regard to jurisdictional claims in published maps and institutional affiliations.

## Supplementary Material

Supplementary Information

## References

[b1] Eckardt K. U. *et al.* Evolving importance of kidney disease: from subspecialty to global health burden. Lancet 382, 158–169 (2013). 23727165 10.1016/S0140-6736(13)60439-0

[b2] Bydash J. R. & Ishani A. Acute kidney injury and chronic kidney disease: a work in progress. Clin. J. Am. Soc. Nephrol. 6, 2555–2557 (2011). 21980184 10.2215/CJN.09560911

[b3] Coca S. G., Singanamala S. & Parikh C. R. Chronic kidney disease after acute kidney injury: a systematic review and meta-analysis. Kidney Int. 81, 442–448 (2012). 22113526 10.1038/ki.2011.379 PMC3788581

[b4] Chawla L. S., Eggersm P. W., Starm R. A. & Kimmel P. L. Acute Kidney Injury and Chronic Kidney Disease as Interconnected Syndromes. N. Engl. J. Med. 371, 58–66 (2014). 24988558 10.1056/NEJMra1214243 PMC9720902

[b5] Tögel F. & Westenfelder C. Recent advances in the understanding of acute kidney injury. F1000Prime Rep. 6, 83 (2014). 25343040 10.12703/P6-83 PMC4166934

[b6] Basile D. P. *et al.* ADQI XIII Work Group: Progression after AKI: Understanding Maladaptive Repair Processes to Predict and Identify Therapeutic Treatments. J. Am. Soc. Nephrol. 27, 687–697 (2016). 26519085 10.1681/ASN.2015030309 PMC4769207

[b7] Basile D. P. *et al.* Identification of persistently altered gene expression in the kidney after functional recovery from ischemic acute renal failure. Am. J. Physiol. Renal. Physiol. 288, F953–F963 (2004). 10.1152/ajprenal.00329.2004 15632414

[b8] Bussolati B. & Camussi G. Therapeutic use of human renal progenitor cells for kidney regeneration. Nat. Rev. Nephrol. 11, 695–706 (2015). 26241019 10.1038/nrneph.2015.126

[b9] Bussolati B. *et al.* Isolation of renal progenitor cells from adult human kidney. Am. J. Pathol. 166, 545–555 (2005). 15681837 10.1016/S0002-9440(10)62276-6 PMC1602314

[b10] Grange C., Moggio A., Tapparo M., Porta S., Camussi G. & Bussolati B. Protective effect and localization by optical imaging of human renal CD133^+^ progenitor cells in an acute kidney injury model. Physiol. Rep. 2, e12009 (2014). 24793983 10.14814/phy2.12009 PMC4098737

[b11] Angelotti M. L. *et al.* Characterization of renal progenitors committed toward tubular lineage and their regenerative potential in renal tubular injury. Stem Cells 30, 1714–1725 (2012). 22628275 10.1002/stem.1130

[b12] Bussolati B., Lauritano C., Moggio A., Collino F., Mazzone M. & Camussi G. Renal CD133^+^/CD73^+^ progenitors produce erythropoietin under hypoxia and prolyl hydroxylase inhibition. J. Am. Soc. Nephrol. 24, 1234–1241 (2013). 23661806 10.1681/ASN.2012080772 PMC3736703

[b13] Moore E. & Bellomo R. Erythropoietin (EPO) in acute kidney injury. Ann. Intensive Care 1, 3 (2011). 21906325 10.1186/2110-5820-1-3 PMC3159901

[b14] Yang F. L., Subeq Y. M., Chiu Y. H., Lee R. P., Lee C. J. & Hsu B. G. Recombinant human erythropoietin reduces rhabdomyolysis-induced acute renal failure in rats. Injury 43, 367–373 (2012). 22209169 10.1016/j.injury.2011.11.013

[b15] Hu L. *et al.* Erythropoietin ameliorates renal ischemia and reperfusion injury via inhibiting tubulointerstitial inflammation. J. Surg. Res. 176, 260–266 (2012). 21816412 10.1016/j.jss.2011.06.035

[b16] Sølling C. *et al.* Erythropoietin administration is associated with short-term improvement in glomerular filtration rate after ischemia-reperfusion injury. Acta Anaesthesiol. Scand. 55, 185–195 (2011). 21226860 10.1111/j.1399-6576.2010.02369.x

[b17] Sharples E. J. *et al.* Erythropoietin protects the kidney against the injury and dysfunction caused by ischemia-reperfusion. J. Am. Soc. Nephrol. 15, 2115–2124 (2004). 15284297 10.1097/01.ASN.0000135059.67385.5D

[b18] Forman C. J., Johnson D. W. & Nicol D. L. Erythropoietin administration protects against functional impairment and cell death after ischaemic renal injury in pigs. BJU Int. 99, 162–165 (2007). 16956351 10.1111/j.1464-410X.2006.06505.x

[b19] Ishii Y. *et al.* Renoprotective effect of erythropoietin against ischaemia-reperfusion injury in a non-human primate model. Nephrol. Dial. Transplant. 26, 1157–1162 (2011). 20935018 10.1093/ndt/gfq601

[b20] Johnson D. W. *et al.* Delayed administration of darbepoetin or erythropoietin protects against ischemic acute renal injury and failure. Kidney Int. 69, 1806–1813 (2006). 16598197 10.1038/sj.ki.5000356

[b21] Okada T., Sawada T. & Kubota K. Asialoeryhtropoietin has strong renoprotective effects against ischemia-reperfusion injury in a murine mode. Transplantation 84, 504–510 (2007). 17713435 10.1097/01.tp.0000277672.02783.33

[b22] Park S. H. *et al.* Erythropoietin decreases renal fibrosis in mice with ureteral obstruction: role of inhibiting TGF-beta-induced epithelial-to-mesenchymal transition. J. Am. Soc. Nephrol. 18, 1497–1507 (2007). 17389738 10.1681/ASN.2005080866

[b23] Imamura R. *et al.* A nonerythropoietic derivative of erythropoietin inhibits tubulointerstitial fibrosis in remnant kidney. Clin. Exp. Nephrol. 16, 852–862 (2012). 22678524 10.1007/s10157-012-0647-x PMC4108904

[b24] Yamaleyeva L. M. *et al.* Cell therapy with human renal cell cultures containing erythropoietin-positive cells improves chronic kidney injury. Stem Cells Transl. Med. 1, 373–383 (2012). 23197816 10.5966/sctm.2011-0048 PMC3659702

[b25] Eliopoulos N., Gagnon R. F., Francois M. & Galipeau J. Erythropoietin delivery by genetically engineered bone marrow stromal cells for correction of anemia in mice with chronic renal failure. J. Am. Soc. Nephrol. 17, 1576–1584 (2006). 16672321 10.1681/ASN.2005101035

[b26] Bussolati B. *et al.* Hypoxia modulates the undifferentiated phenotype of human renal inner medullary CD133^+^ progenitors through Oct4/miR-145 balance. Am. J. Physiol. Renal. Physiol. 302, F116–F128 (2012). 21900452 10.1152/ajprenal.00184.2011

[b27] Ang D. H. *et al.* Impaired angiogenesis in the remnant kidney model. I. Potential role of vascular endothelial growth factor and thrombospondin-1. J. Am. Soc. Nephrol. 12, 1434–1447 (2001). 11423572 10.1681/ASN.V1271434

[b28] Basile D. P. *et al.* Impaired endothelial proliferation and mesenchymal transition contribute to vascular rarefaction following acute kidney injury. Am. J. Physiol. Renal Physiol. 300, F721–F733 (2001). 10.1152/ajprenal.00546.2010 PMC3064142 21123492

[b29] Lazzeri E. *et al.* Regenerative potential of embryonic renal multipotent progenitors in acute renal failure. J. Am. Soc. Nephrol. 18, 3128–3138 (2007). 17978305 10.1681/ASN.2007020210

[b30] Kumar S. *et al.* Sox9 activation highlights a cellular pathway of renal repair in the acutely injured mammalian kidney. Cell Rep. 12, 1325–1338 (2015). 26279573 10.1016/j.celrep.2015.07.034

[b31] Giglio M. J., Bozzini C. E., Barcat J. A. & Arrizurieta E. Relationship between severity of renal damage and erythropoietin production in uranyl nitrate-induced acute renal failure. Exp. Hematol. 14, 257–260 (1986). 3699109

[b32] Giglio M. J., Huygens P., Frid A., Bozzini C. E., Barcat J. A. & Arrizurieta E. Depressed plasma erythropoietin levels in rats with hemodynamically-mediated acute renal failure. Acta Physiol. Pharmacol. Latinoam. 40, 299–308 (1990). 2094164

[b33] Nielsen O. J. & Thaysen J. H. Erythropoietin deficiency in acute renal failure. Lancet 1, 624–625 (1989). 10.1016/s0140-6736(89)91662-0 2564155

[b34] Lipkin G. W., Kendall R. G., Russon L. J., Turney J. H., Norfolk D. R. & Brownjohn A. M. Erythropoietin deficiency in acute renal failure. Nephrol. Dial. Transplant. 5, 920–922 (1990). 2127826 10.1093/ndt/5.11.920

[b35] Liangos O., Pereira B. J. & Jaber B. L. Anemia in acute renal failure: Role for erythropoiesis-stimulating proteins? Artif. Organs 27, 786–791 (2003). 12940900 10.1046/j.1525-1594.2003.07287.x

[b36] La Ferla K., Reimann C., Jelkmann W. & Hellwig-Bürgel T. Inhibition of erythropoietin gene expression signaling involves the transcription factors GATA-2 and NF-kappaB. FASEB J. 16, 1811–1813 (2002). 12223449 10.1096/fj.02-0168fje

[b37] Obara N., Suzuki N., Kim K., Nagasawa T., Imagawa S. & Yamamoto M. Repression via the GATA box is essential for tissue specific erythropoietin gene expression. Blood 111, 5223–5232 (2008). 18202227 10.1182/blood-2007-10-115857

[b38] Souma T. *et al.* Plasticity of Renal Erythropoietin-Producing Cells Governs Fibrosis. J. Am. Soc. Nephrol. 24, 1599–1616 (2013). 23833259 10.1681/ASN.2013010030 PMC3785278

[b39] Chang Y. T. *et al.* DNA methyltransferase inhibition restores erythropoietin production in fibrotic murine kidneys. J. Clin. Invest. 126, 721–731 (2016). 26731474 10.1172/JCI82819 PMC4731189

[b40] Horiguchi H., Oguma E. & Kayama F. Cadmium and cisplatin damage erythropoietin-producing proximal renal tubular cells. Arch. Toxicol. 80, 680–686 (2006). 16555044 10.1007/s00204-006-0093-1

[b41] Zhao C., Lin Z., Luo Q., Xia X., Yu X. & Huang F. Efficacy and Safety of Erythropoietin to Prevent Acute Kidney Injury in Patients With Critical Illness or Perioperative Care: A Systematic Review and Meta-analysis of Randomized Controlled Trials. J. Cardiovasc. Pharmacol. 65, 593–600 (2015). 26065644 10.1097/FJC.0000000000000229 PMC4461384

[b42] Breggia A. C., Wojchowski D. M. & Himmelfarb J. JAK2/Y343/STAT5 signaling axis is required for erythropoietin-mediated protection against ischemic injury in primary renal tubular epithelial cells. Am. J. Physiol. Renal. Physiol. 295, F1689–F1695 (2008). 18815218 10.1152/ajprenal.90333.2008 PMC2753302

[b43] Ribatti D. Angiogenic effects of erythropoietin. Int, Rev. Cell Mol. Biol. 299, 199–234 (2012). 22959304 10.1016/B978-0-12-394310-1.00005-9

[b44] Lv W., Li W. Y., Xu X. Y., Jiang H. & Bang O. Y. Bone marrow mesenchymal stem cells transplantation promotes the release of endogenous erythropoietin after ischemic stroke. Neural. Regen. Res. 10, 1265–1270 (2015). 26487854 10.4103/1673-5374.162759 PMC4590239

[b45] Bi B., Guo J., Marlier A., Lin S. R. & Cantley L. G. Erythropoietin expands a stromal cell population that can mediate renoprotection. Am. J. Physiol. Renal Physiol. 295, F1017–F1022 (2008). 18653480 10.1152/ajprenal.90218.2008 PMC2576137

[b46] Lacombe C. *et al.* Peritubular cells are the site of erythropoietin synthesis in the murine hypoxic kidney. J. Clin. Invest. 81, 620–623 (1988). 3339134 10.1172/JCI113363 PMC329613

[b47] Maxwell P. H. *et al.* Identification of the renal erythropoietin-producing cells using transgenic mice. Kidney Int. 44, 1149–1162 (1993). 8264149 10.1038/ki.1993.362

[b48] Kobayashi H. *et al.* Distinct subpopulations of FOXD1 stroma-derived cells regulate renal erythropoietin. J. Clin. Invest. 126, 1926–1938 (2016). 27088801 10.1172/JCI83551 PMC4855934

[b49] Nagai T. *et al.* Reevaluation of erythropoietin production by the nephron. Biochem. Biophys. Res. Commun. 449, 222–228 (2014). 24832733 10.1016/j.bbrc.2014.05.014

[b50] Smeets B. *et al.* Proximal tubular cells contain a phenotypically distinct, scattered cell population involved in tubular regeneration. J. Pathol. 229, 645–659 (2013). 23124355 10.1002/path.4125 PMC3951144

[b51] Romagnani P. & Remuzzi G. CD133^+^ renal stem cells always co-express CD24 in adult human kidney tissue. Stem Cell Res. 12, 828–829 (2014). 24467938 10.1016/j.scr.2013.12.011

[b52] Tian X., Woll P. S., Morris J. K., Linehan J. L. & Kaufman D. S. Hematopoietic engraftment of human embryonic stem cell-derived cells is regulated by recipient innate immunity. Stem Cells 24, 1370–1380 (2006). 16456127 10.1634/stemcells.2005-0340

[b53] Dame C. *et al.* Hepatic erythropoietin gene regulation by GATA-4. J. Biol. Chem. 279, 2955–2961 (2004). 14583613 10.1074/jbc.M310404200

