# Supplementary material for: Human CD133+ Renal Progenitor Cells Induce Erythropoietin Production and Limit Fibrosis After Acute Tubular Injury
Source: Sci Rep. 2016 Nov 17;6:37270. doi: 10.1038/srep37270 (PMC5112528; doi:10.1038/srep37270)
Supplement: Supplementary Information [file srep37270-s1.pdf]

HUMAN CD133^{+} RENAL PROGENITOR CELLS INDUCE ERYTHROPOIETIN PRODUCTION AND LIMIT FIBROSIS AFTER ACUTE TUBULAR INJURY

Shikhar Aggarwal[1], Cristina Grange[2], Corinne Iampietro[1], Giovanni Camussi[2] and Benedetta Bussolati[1]

[1]Department of Biotechnology and Health Sciences, Molecular Biotechnology Center and [2]Department of Medical Sciences, Molecular Biotechnology Center; University of Turin, Italy.

Supplementary Files

Supplementary Figure 1

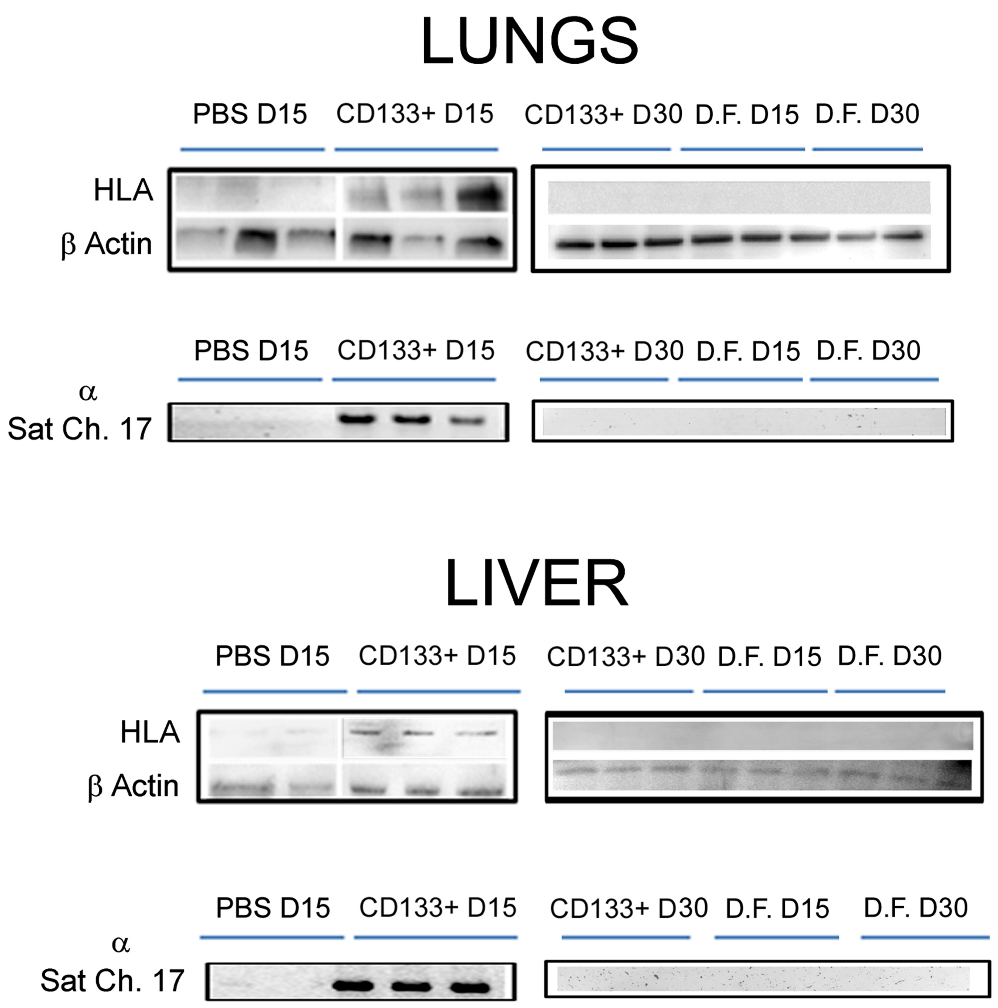

Localization of intravenously injected CD133^{+} cells in lungs and liver of AKI SCID mice. HLA protein and whole genome DNA analysis (α-Sat ch17) of mice lungs and liver at day 15 and day 30 after glycerol injection. CD133^{+} cells were present up to day 15 in lungs and liver as shown by protein /DNA analysis as compared to dermal fibroblasts (D.F.), which were not detected at day 15. No cells (CD133^{+} or fibroblasts) were detected at day 30. Lanes run on different gels are separated by a dark line.

Supplementary Figure 2

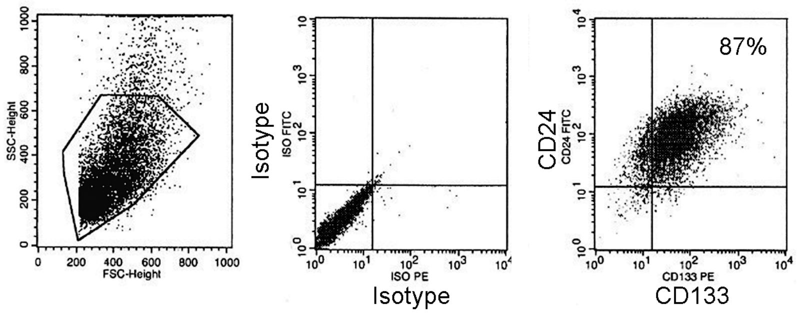

Representative cytofluorimetric analysis of the expression of CD133 and CD24 in the cell preparations in study. All cell lines were characterized and showed co-expression of CD133 and CD24 in >85% cells.

Supplementary Table I. Phenotypic characteristics of CD133^{+} cells.

**Markers**	**CD133**	**CD24**	**CD73**	**CD44**	**CD90**	**CD29**	**Vimentin**	**Pax2**
% positive cells	96.0	87.8	96.5	96.8	77.3	81.9	>95	>95

The expression of the renal progenitor markers CD133, CD24 and Pax2 and of the mesenchymal markers CD73, CD44, CD90, CD29 and vimentin by CD133^{+} cell isolates used in the study was evaluated by cytofluorimetric analysis or immunofluorescence staining, as described. Cells were tested between the second and the third culture passage. Data are mean of four different cells lines.
